# Knowledge of community care workers about key family practices in a rural community in South Africa

**DOI:** 10.4102/phcfm.v7i1.892

**Published:** 2015-12-17

**Authors:** Ethelwynn Stellenberg, Marjorie van Zyl, Johanna Eygelaar

**Affiliations:** 1 Division of Nursing, Stellenbosch University, South Africa

## Abstract

**Background:**

Interventions by community care workers within the context of community-based integrated management of childhood illness (CIMCI) may have a positive effect on child health if the health workers have adequate knowledge about key family practices.

**Setting:**

The study was conducted in rural areas of the West Coast district in the Western Cape, South Africa.

**Objectives:**

The objective of this study was to determine the knowledge of community care workers about five of the 16 key family practices of CIMCI.

**Methods:**

A descriptive survey collected a self-administered questionnaire from 257 community care workers out of a possible total of 270 (95.2% response rate). Descriptive and inferential statistical analysis was applied.

**Results:**

Only 25 of the respondents (10%) obtained a score higher than 70% on the knowledge-based items of the questionnaire. Less than 25% of respondents answered questions in these key areas correctly (pneumonia [17%], tuberculosis [13%], HIV/AIDS [9%] immunisation [3%] and recommendations for a child with fever [21%]). Statistically significant correlations were found between the total score a respondent achieved and the highest level of education obtained (*p* < 0.01), the level of in-service training (p < 0.01), attendance of a CIMCI five-day training course (*p* < 0.01), and completing a subsequent refresher course (*p* < 0.01).

**Conclusion:**

The knowledge of CCWs was inadequate to provide safe, quality CIMCI. CIMCI refresher courses should be offered annually to improve CCWs’ knowledge and the quality of care that they render. Regular update courses could contribute to building competence.

## Abstrait

**Les connaissances des prestataires de soins communautaires sur les pratiques familiales clés dans une communauté rurale d’Afrique du Sud.**

**Contexte:**

Les interventions par des prestataires de soins communautaires dans le contexte de la gestion intégrée communautaire des maladies de l’enfance (GICME) peuvent avoir un effet positif sur la santé des enfants si les prestataires de soins ont suffisamment de connaissances sur les pratiques familiales clés.

**Lieu:**

L’étude a été menée dans les régions rurales de la Cote Ouest du Western Cape, en Afrique du Sud.

**Objectifs:**

L’objectif de cette étude était de déterminer les connaissances des prestataires de soins communautaires (PSC) sur cinq des 16 pratiques familiales clés de la GICME.

**Méthodes:**

Un plan de recherche descriptif et exploratoire avec approche quantitative a été utilisé. On a obtenu un taux de réponse de 95.2% de la population ciblée (N = 270). On a collecté les données au moyen d’un questionnaire auto-administré. On a utilisé une analyse statistique descriptive et différents tests statistiques.

**Résultats:**

Seuls 25 des répondants (10%) ont obtenu un score supérieur à 70% sur les questions du questionnaire basées sur les connaissances. Les connaissances des PSC étaient très faibles dans le domaine de la morbidité élevée et des taux de mortalité des enfants. Moins de 25% des répondants ont répondu correctement aux questions dans ces domaines clés (pneumonie [17%], tuberculose [13%], VIH/SIDA [9%] immunisation [3%] et recommandations pour un enfant fiévreux [21%]). On a trouvé des corrélations statistiquement importantes entre le score total d’un répondent et le plus haut niveau d’éducation atteint (*P* < 0.01), le niveau de formation en cours d’emploi (*P* < 0.01), assistance à un cours de formation de cinq jours sur la GICME (*P* < 0.01), et l’achèvement ultérieur d’un cours de rafraichissement (*P* < 0.01).

**Conclusion:**

Il faut offrit tous les ans des cours de rafraichissement sur la GICME pour améliorer les connaissances des PSC et la qualité des soins qu’ils donnent. Des cours de mise à jour réguliers pourraient permettre de développer les compétences.

## Introduction

### Social value

With an estimated 8.1 million children under the age of five years dying every year, child mortality is a worldwide problem,^1^ despite the fourth Millennium Development Goal of the World Health Organization (WHO) aiming to reduce child mortality by two-thirds by 2015.^2^ Almost 90% of the deaths in this age group are attributed to neonatal causes, pneumonia, diarrhoea, malaria, measles and HIV and/or AIDS.^3^

An integrated approach that focuses on the holistic well-being of the child, namely integrated management of childhood illness, aims to improve care at first-level health facilities by introducing standardised training for health workers and treatment guidelines.^4^

In 1997, the WHO and the United Nations Children’s Fund (UNICEF) identified 16 key family practices that could be included in a community-based approach to integrated management of childhood illness (CIMCI).^5^ Studies have shown that interventions by community care workers (CCWs) who follow the CIMCI approach reduce morbidity and mortality in children under the age of five^6,7^ and may contribute to achieving the Millennium Development Goals.^5^

CCWs represent the communities that they serve and are involved in outreach in a variety of community settings.^8^ However, CCW training and refresher courses are not standardised and the care workers’ knowledge about CIMCI may be inadequate.

### Scientific value

A study conducted in Ile-Ife in south-western Nigeria has shown that well-implemented CIMCI interventions offer an effective, low-cost approach to ensuring optimal growth, development and survival of Nigerian children.^9^ CCWs use their skills to ensure that their clients receive the necessary services through capacity building and advocating the importance of a healthy lifestyle to ensure good health.^8^ Explaining the importance of rehydration therapy and promoting exclusive breastfeeding are two important health-promoting interventions by CCWs that could lower the mortality of children under the age of five by 10%.^10^

The results of the Nigerian study showed that 78.5% of caregivers from CIMCI-compliant local government areas of Osun, an inland state in south-western *Nigeria,* obtained information about the home management of childhood illnesses from trained CCWs.^9^ In contrast, about two-thirds (67.9%) of caregivers from non-compliant local government areas received information about the home management of childhood illnesses from relatives. The study further showed that in CIMCI-compliant local government areas, the use of an oral rehydration solution for the management of childhood diarrhoea was high (90.8%), whereas it was below 20% in non-compliant local government areas. In the CIMCI-compliant areas the recommended treatment for diarrhoea was a salt-sugar solution, together with the use of antibiotics and referral to a primary healthcare centre; in the areas that did not follow the CIMCI approach, the caregivers used home remedies such as water, garri water and salt water, and patients were not referred to a primary healthcare facility. After CIMCI implementation, the prevalence of diarrhoea was reduced from 13% to 9.5%.^9^

### Training of CCWs

CCWs need to be trained appropriately to ensure that health information is disseminated effectively in their communities. A study in Kenya found that CCWs often misinterpreted signs and symptoms of common diseases, resulting in improper drug management.^11^ Consequently, as one of the CIMCI interventions, CCWs were provided with specific guidelines for the treatment of pneumonia and malaria in remote areas. In a study conducted in India, poor training of CCWs revealed that only 12.8% of mothers knew that the Bacillus Calmette-Guerin vaccine is given at birth and that only 6% knew that the hepatitis B vaccine could also be given at birth. Consequently less than half (37.2%) of the mothers in the study had their children immunised according to the immunisation schedules for their children by the age of nine months.^12^ The selection, retention and training of CCWs are thus critical factors for the success of India’s immunisation programme.^13^

Kenyan CCWs are required to be able to read at school level (grade 9 or higher) and to volunteer in the community they live in. Women are preferred as CCWs. Candidates to be trained as CCWs are selected by health committees that are established in some communities. Remuneration for their services, recognition from the community, a feeling of selfsatisfaction and appreciation expressed by the community have been cited as incentives for CCWs to adhere to their work.^11^

In the United States, 91% of CCWs are women and 37.6% are younger than 50 years. They work at private homes, community centres, clinics and hospitals. The most common health issues addressed by CCWs are HIV and/or AIDS, women’s health, pre- and postnatal care, child health and cancer. CCWs assist their clients with access to both medical and nonmedical services, provide culturally appropriate health education and information, are involved in community advocacy and provide social support.^14^

Training and ongoing supervision of CCWs vary widely across the world. In ten Latin American countries where communities use training materials developed by the Red Cross, the recommended practices were adapted for local relevance.^15^ This led to 29% more families recognising the warning signs of diarrhoea, 31% more families knowing the danger signs of illness in a child and the consequences of not taking action, and 12% more families being able to recognise the danger signs of pneumonia. Most notably, 6% more children with a cough accompanied with rapid breathing were taken to health services.

In the Siaya district in Kenya, CCWs received training on how to use the guideline for managing a sick child.^11^ The initial training included ten days of lectures, during which case studies were reviewed and participants engaged in role-playing, and five days of clinical practice at the Siaya District Hospital. Refresher training sessions to address targeted weaknesses in clinical skills were offered for six to fifteen days. In addition to lectures and roleplaying, CCWs’ training involved reviewing videotaped consultations and clinical practice at local pharmacies or health facilities in groups of eight to ten. The CCWs received medicine kits to treat children diagnosed with malaria or pneumonia or who experienced diarrhoea or fever and suffered from malnutrition. Overall adherence to clinical guidelines was fairly acceptable, with, on average, 80% of all guideline-recommended procedures being performed correctly.^11^

CCWs in the USA commonly receive training on cultural awareness, knowledge of health issues and social services, interpersonal communication skills and client advocacy. The least common topic is leadership. Carers who wish to qualify as CCWs can obtain a diploma of education and training offered by a prestigious local university.^14^ In contrast, no specific, formal training is offered to CCWs in Mexico, although they can attend educational workshops once or twice a month to develop their skills.^16^

In southern Asia, a study from Sylhet, Bangladesh, showed that CCWs working in rural districts received 21 days of training with regard to newborn breastfeeding and a practical session on the observation and assessment of breastfeeding.^17^ Training included lectures, hands-on demonstration and practical exercises with the help of post-partum breastfeeding mothers, and video-guided lessons with support from a supervisor. In nearby Nepal, women with basic school education, as well as those who are only semi-literate or illiterate, have been trained as CCWs to diagnose and assess severity and danger signs associated with acute respiratory infection or diarrhoea in children, offer appropriate treatment and refer patients to health facilities.^18^ As part of this initiative, the WHO–developed curriculum for integrated management of childhood illnesses was adapted and simplified to ensure that it met the needs of these volunteers.^18^ In this rural district under study, the services of CCWs follow an integrated approach, which includes home visits to render basic nursing care, CIMCI, directly observed tuberculosis treatment, adherence support for clients on antiretroviral therapy and services for managing mental illness and chronic lifestyle diseases. According to this model, the same CCW renders the full package of care, whereas in the urban model different CCWs are responsible for the respective programmes.^19^

## Aim of the study

Between 2009 and 2011, the infant mortality rate in the study area (West Coast district) was approximately 30/1000 live births, with the mortality rate amongst children younger than five years approximately 35/1000 live births. The five most common causes of death in this population were pneumonia, premature birth, diarrhoea, HIV and/or AIDS and various injuries.^20^

It was therefore deemed essential that CCWs’ knowledge of key family practices be explored. Five specific practices were identified, namely breastfeeding, physical growth and development, common childhood illnesses, immunisations and danger signs for referral to a clinic or hospital. These danger signs in children include inability to drink or breastfeed, vomiting, lethargy or unconsciousness, coughing accompanied with rapid breathing (> 50 breaths per minute), coughing associated with chest indrawing, bloody diarrhoea, diarrhoea together with presentation of sunken eyes or a sunken fontanel, and sudden fever in a child younger than two months or who is not feeding properly.

## Research methods and design

### Study design

The study involved a descriptive, quantitative survey of CCWs’ knowledge with regard to five of the 16 key family practices described for the CIMCI approach.

### Setting

The study was conducted in the West Coast district, which is subdivided into five sub-districts (Swartland, Bergriver, Saldanha, Cederberg and Matzikama). At the time of the research, community-based services were rendered by 17 non-profit organisations, which employed 270 CCWs to serve a total of 12 000 clients. Table 1 shows the distribution of CCWs across the sub-districts.

**TABLE 1:** Distribution of community care workers across the sub-districts of the West Coast district, Western Cape, South Africa.

Sub-districts in the West Coast district	Number of CCWs per sub-district
Matzikama	90
Cederberg	54
Swartland	50
Bergriver	33
Saldanha	43
**Total**	**270**

CCW, community care worker.

### Study population sampling strategy

The target population (N = 270) consisted of CCWs working for non-profit organisations funded by the provincial government and who provided CIMCI in the West Coast district of the Western Cape, South Africa. All CCWs were invited to participate in the study; those who refused consent or who were not available during the two weeks of data collection (owing to, for example, leave) were excluded from the study.

### Data collection

All data were collected in 2012. One of the researchers collected the data from a self-administered questionnaire that included 25 knowledge-based items related to the five key family practices identified for this study. The questionnaire was designed to be completed within 30 minutes and was available in either English or Afrikaans. In addition to the knowledge-based section, the questionnaire also included questions related to demographic data (respondent’s age; highest level of education; gender; health-related work experience; experience working with children; working hours; value of the respondent’s stipend).

A variety of open- and closed-ended questions, graphs and pictures were used in the questionnaire. As the CIMCI programme has no specific score for competence, a marking schedule was designed for this study together with primary health care experts from the Division of Nursing, Stellenbosch University. The minimum score for safe, quality care was set at 70%.

#### Pilot study

A pilot study was conducted in the Bergriver sub-district to test the methods and the instrument to be used in the main investigation. The pilot study included 33 CCWs, representing 12.2% of the respondents of the main study.

The pilot study showed that the researcher needed to be present to give contextual structure and guide respondents during their completion of the questionnaire. This was to ensure that all the questions were completed. The questionnaire was found to be easily understood and that the allocated time was adequate for completion.

#### Reliability and validity

Content, construct and face validity were ensured through consultation with experts in primary health care, research methodology and statistics. Senior researchers (study supervisors), the post-graduate committee of Stellenbosch University and ethical committees of various relevant institutions approved the study design and approach. The content of the questionnaire was based on the 16 key family practices identified for inclusion in a CIMCI programme. Reliability and validity were further improved by the researcher personally collecting and capturing the data, thereby ensuring a consistent data collection process. A pilot study was also conducted.

### Data analyses

Data were analysed using statistical software (STATISTICA 10, StatSoft Inc., Tulsa). Descriptive statistical tests (Spearman rank order and Mann-Whitney *U* correlation) were used to determine the level of significance between the dependent and independent variables at a 95% confidence level. The level for statistical significance was defined as *P* < 0.05.

## Ethical considerations

Ethical approval was received from the Human Research Ethics Committee, Stellenbosch University, and permission from the Provincial Government of the Western Cape, and the West Coast District Department of Health.

Participation was entirely voluntary and refusal to participate was without consequence. A respondent was free to leave the study at any time, even after having agreed to take part. Informed written consent was obtained from each respondent. The purpose, procedures, risks and benefits of the research project, as well as the obligations and commitments of both the participants and the researcher, were outlined in the consent form. To ensure confidentiality and anonymity, the consent form was submitted separately from the questionnaire. The collected data were managed only by the researchers.

The right to protection from discomfort and harm is based on the ethical principle of beneficence, which dictates that participation in the study should not cause any harm to the respondent.^21^ The information obtained during the study was not of sensitive nature and no known risks to the respondents were foreseen during the study. The respondents were protected from psychological harm, but would have been referred to appropriate psychological services in the event of any psychological harm.

The selection of respondents was deemed fair as all of the CCWs working in the study area were invited to take part in the study. This population of CCWs were chosen to participate in the study because they were connected to the research problem. The only participants who were excluded from the study were those not physically present during data collection and those who refused consent. Benefits derived from participating in the study were communicated to the participants and to the relevant authorities.

## Results

A total of 257 CCWs completed the questionnaire (response rate, 95.2%). The most common age group amongst the respondents was 36–40 years (n = 55; 21%). The majority of respondents (n = 252; 98%) were female and close to half (n = 116; 45%) had passed grade 12 schooling. Approximately half of the respondents (n = 130; 51%) indicated that they had not received CIMCI training, despite providing services for an average of six years already; 124 respondents (48%) had received some training between 2004 and 2012, of which 58 attended a refresher course in 2012. Respondents indicated that they were expected to provide CIMCI according to a community-based model in which up to 28 clients had to be attended to per day. The mean and median number of clients attended to per day were 11 and 12, respectively.

Total scores for the 25 knowledge-based questions included in the questionnaire show that 87 of the respondents (34%) scored below 50%. The majority of the respondents scored between 50% and 69% (n = 145; 56%) and only 25 respondents (10%) scored between 70% and 89%. None of the participants scored 90% or more (Table 2).

Results of the Spearman’s rank order test applied to the investigated variables are shown in Table 3. A positive correlation (Spearman rho = 0.1) was found between the highest school grade passed and the total knowledge score of respondents (*P* < 0.01) (Table 3). A positive correlation with a medium effect (Spearman rho = 0.3) was also found between having received training as part of the Expanded Public Works Programme (EPWP) and an individual’s total scores (*P* = 0.01). A medium effect relationship ranges between *r* = 0.3 and *r* = 0.49. Additional EPWP training was associated with higher knowledge scores. The EPWP is a nationwide programme aimed at integrating the unemployed into productive work by providing learners with in-service skills training and so allows them to earn an income through a stipend.

Results of the Mann-Whitney U-test indicated a significant relationship between the total knowledge score obtained and attending a five-day CIMCI training course (*P* < 0.01) and later a refresher course (*P* < 0.01).

Table 4 shows the number of participants who correctly answered the questions in the key knowledge areas. Knowledge about managing conditions associated with high mortality and morbidity rates were especially poor. These included pneumonia (17%), tuberculosis (13%) and HIV and/or AIDS (9%), correct immunisation at six weeks (3%), recommendations for a child with fever (21%) and all danger signs (5%).

**TABLE 2:** Distribution of test scores with regard to knowledge of community-based integrated management of childhood illness (N = 257).

Performance category (score range)	Number of respondents *n (%)*
Poor (0%-49%)	87 (34)
Average (50%-69%)	145 (56)
Good (70%-89%)	25 (10)
Excellent (90%-100%)	0 (0)

**TABLE 3:** Relationships between the total scores and biographical data.

Variables	*N*	Test	Spearman *r*	P-value
Total score and EPWP	257	Spearman rho	0.3	< 0.01
Total score and age	257	Spearman rho	0.07	0.2
Total score and highest school grade passed	255	Spearman rho	0.19	< 0.01

EPWP; Expanded Public Works Programme.

**TABLE 4:** Number of respondents who answered questions about key family practices associated with high mortality and morbidity rates correctly (N = 257).

Knowledge item	Number of respondents who answered correctly n (%)
Only one sign to suspect pneumonia	45 (17)
Tuberculosis	34 (13)
HIV and/orAIDS	23 (9)
Correct immunisation at 6 weeks	7 (3)
Recommendations for child with fever	55 (21)
All danger signs	12 (5)

## Discussion

Only 10% of the participants scored above the defined minimum score for quality care, with 34% of respondents scoring below 50%. The respondents’ knowledge regarding the selected aspects of family health included in the 16 key family practices of CIMCI should be associated with scores ≥ 70%. Inadequate and incorrect knowledge may have an influence on child mortality and child morbidity in the communities where the CCWs work.

Scores on questions related to breastfeeding showed that respondents’ general knowledge was only of an average standard. The most important potential advantage of exclusive breastfeeding for six months relates to the prevention of infectious diseases, especially morbidity and mortality related to gastrointestinal infections.^22^ In addition, women stay in hospital only for a short period after giving birth and therefore health promotion on breastfeeding at a community level is very important.^23^

Respondents’ knowledge of managing pneumonia, tuberculosis and HIV/AIDs was poor. If not diagnosed early, these conditions increase infant and child morbidity and mortality. The five most common causes of death of children under five of this region are pneumonia, prematurity, diarrhoea, HIV and/or AIDS, and injuries.^20^ The respondents’ knowledge was not adequate to prevent and manage diarrhoea in this region under study. If a CCW does not know how to prepare the sugar–salt solution to be administered during diarrhoea (as enquired about in the questionnaire), child morbidity and mortality could increase unnecessarily owing to dehydration. Diarrhoeal disease is the second leading cause of death in children younger than five years. This condition is both preventable and treatable.^24^

Results also showed that respondents’ knowledge about immunisation and recommendations for treating a child with fever was poor. Ten danger signs (as described earlier) have been identified for referring children to a clinic or hospital and awareness of these is important for early referral to reduce or prevent child morbidity and mortality.^25^

Knowledge about immunisation is prioritised on the Road to Health card^26^ that a CCW has to evaluate during a routine household visit. An Indian study showed that less than half (37.2%) of mothers completed routine immunisation schedules for their children by the age of nine months owing to poor knowledge.^12^

Studies have shown that the CIMCI approach can reduce morbidity and mortality in children under the age of five.^6,7^ The literature reflects a great diversity of approaches to locations, organisation and length of training. However, if refresher training is not available, the skills and knowledge acquired in service are lost quickly.^27^ The curriculum for the CIMCI course therefore needs to be standardised by the National Department of Health to ensure better quality assurance of training. In this survey, 51% of CCWs have been practising for six years without any applicable training, with refresher courses being attended at random. A large proportion of the surveyed CCWs (61%) have never attended a refresher course. It is mandatory that the community-based service model is practised in the West Coast region,^28^ which requires CCWs to manage adult clients as well. Almost half of the respondents (47%) indicated that they see between 11 and 15 clients per day, with 11% indicating that they attend to more than 15 clients per day.

### Strengths and limitations

Although this study was conducted in only one district (West Coast district) of the province, it reached the full population of CCWs in the five sub-districts and should be a valid and reliable picture of their knowledge of CIMCI. The findings are likely to be generalisable to the rest of the province, especially the other rural districts, which are all managed under a single chief director, have the same model of contracting CCWs and similar populations. A limitation identified was that this study specifically targeted a rural region and excluded urban areas.

### Implications and recommendations

To reduce infant morbidity and mortality rates, selection criteria should be introduced to recruit suitable candidates to train as CCWs. This is supported by our results, which showed a positive correlation between advanced schooling and achieving high knowledge scores.

Knowledge should be improved, which, given the working context of CCWs, should be based on competence and practice. Training materials and activities should be developed specifically for CCWs and tailored to the literacy level of the CCWs. CIMCI refresher courses should be offered annually and these need to be standardised and implemented to strengthen CCWs’ knowledge and the quality of care that they render. Regular monthly update courses could contribute to shaping competent CCWs with appropriate knowledge and skills to serve their community.

## Further research

It is recommended that the training being offered to CCWs should be evaluated, including in urban settings where the model of community health work may differ. Interventions should be introduced and re-evaluated scientifically.

## Conclusion

This article described an investigation into the knowledge of the CIMCI programme amongst CCWs working in a rural district in the Western Cape, South Africa. Results showed that the knowledge base of a third of the participants was poor and only 10% had sufficient knowledge to provide safe, quality care. Higher levels of basic education and all forms of training in CIMCI were associated with better knowledge scores. Standardised training is essential for delivering effective CIMCI and with the use of an appropriate curriculum CCWs could be trained and deployed within a year.^29^
